# Chemotherapy for the treatment of malignant peripheral nerve sheath tumors in neurofibromatosis 1: a 10-year institutional review

**DOI:** 10.1186/1750-1172-8-127

**Published:** 2013-08-23

**Authors:** Ouidad Zehou, Elizabeth Fabre, Laurent Zelek, Emilie Sbidian, Nicolas Ortonne, Eugeniu Banu, Pierre Wolkenstein, Laurence Valeyrie-Allanore

**Affiliations:** 1 Department of Dermatology, Referral center for Neurofibromatosis, Henri Mondor Hospital, UPEC, Créteil, France; 2 Department of Medical Oncology, Georges Pompidou European Hospital, Paris, France; 3 Department of Medical Oncology, Avicenne Hospital, Bobigny, France; 4 Department of Pathology, Henri Mondor Hospital, UPEC, Créteil, France; 5 Department of Medical Oncology, Cancer Institute “Ion Chiricuta”, Cluj-Napoca, Romania; 6 Department of Dermatology, Henri-Mondor Hospital, 51 Av du Maréchal de Lattre de Tassigny, F-94010 Créteil Cedex, France

**Keywords:** Neurofibromatosis 1, Malignant peripheral nerve sheath tumor, Soft tissue sarcoma, Chemotherapy

## Abstract

**Background:**

Neurofibromatosis 1 (NF1) is the most common autosomal dominant disorder, with an incidence of 1 in 2,500-3,300 live births. NF1 is associated with significant morbidity and mortality because of complications, especially malignant peripheral nerve sheath tumors (MPNSTs), which mainly develop during adulthood. We evaluated our experience with management of NF1 with MPNSTs by standard chemotherapy with anthracycline and/or ifosfamide in terms of time to treatment failure and overall survival.

**Methods:**

We performed a retrospective review of consecutive patients with NF1 and a diagnosis of MPNSTs between 1993 and 2003 in our referral center for NF1. Prognostic factors were evaluated by univariate analysis.

**Results:**

We evaluated data for 21 patients with grade 1 (n=1), grade 2 (n=8) and grade 3 (n=12) MPNST; 16 presented localized disease and underwent surgery: margins for 6 were tumor-free (including 3 patients with amputation), 2 showed microscopic residual disease and 8 showed macroscopic residual disease. All patients received chemotherapy and 9 radiotherapy. Median time to treatment failure and overall survival were 7.8 and 17 months, respectively. Two patients were still alive at 138 and 167 months. We found no significant relationship between type of chemotherapy and time to treatment failure or overall survival.

**Conclusions:**

MPNSTs are highly aggressive in NF1. Conventional chemotherapy does not seem to reduce mortality, and its role must be questioned. Recent advances in the molecular biology of MPNSTs may provide new prognostic factors and targeted therapies.

## Background

Malignant peripheral nerve sheath tumors (MPNSTs) are uncommon, representing about 5% of soft-tissue sarcomas. Neurofibromatosis 1 (NF1) is one of the most common autosomal dominant disorders, with an incidence of 1 in 2,500-3,300 live births. It is associated with mutation in *Nf1*, a tumor suppressor located on chromosome 17q11.2. *Nf1* encodes neurofibromin, a protein of the *ras* signal transduction pathway [1]. NF1 is characterized by neurofibromas, *café au lait* spots, intertriginous freckling, bone malformations, learning disabilities and iris hamartomas [1].

NF1 has a significant morbidity and mortality because of various complications, especially benign and/or malignant tumors. Neurofibromas are benign tumors mostly composed of Schwann cells, perineurium like-cells, fibroblasts and mast cells. Cutaneous neurofibromas greatly affect quality of life; subcutaneous, nodular and internal neurofibromas act mainly through compression and can transform into MPNSTs. Several clinical features such as internal or subcutaneous neurofibromas are predictors of mortality with NF1 [1,2]. Patients with subcutaneous neurofibromas are 3 times more likely to have internal plexiform neurofibromas and MPNSTs. In those with internal plexiform neurofibromas, MPNSTs are 20 times more likely to develop [1]. The overall risk of cancer is more than three-fold greater than in the general population, and MPNSTs are the leading cause of death during adulthood [3,4]. Hence, the lifetime risk of MPNSTs is about 8% to 13% [5-8].

An enlarged mass, neurological deficits and pain can predict malignant transformation of MPNSTs [9]. These NF1 MPNSTs are associated with poor prognosis; the 5-year survival rate is between 16% and 38% [7,10-15]. Limited disease is treated by wide excision along with radiation therapy for high-risk tumors defined as intermediate- to high-grade deep tumors with a diameter >5 cm [16]. Adjuvant chemotherapy is not standard treatment in adult-type soft-tissue sarcomas and can be proposed for high-risk tumors. Extensive disease is treated with anthracycline-based chemotherapy. Ifosfamide may be discussed for patients with good performance status [16].

Here we retrospectively examined our experience with the management of MPNSTs by standard chemotherapy with anthracycline and/or ifosfamide in patients with NF1 and evaluated prognostic factors of time to treatment failure and overall survival.

## Methods

### Patients

We included data for all consecutive NF1 patients with a diagnosis of MPNST between February 1993 and November 2003 who underwent chemotherapy in our institution, a French national referral center for neurofibromatoses. Data on clinico-pathological features and other variables were collected from medical charts and included medical history; demographic characteristics (gender, age); clinical presentation, including pain, motor or sensitive deficits, tumor location and size (the largest diameter assessed by CT or MRI before surgery and/or chemotherapy); intraoperative and macroscopic pathological evaluation; metastatic status; histopathological grade of the primary tumor according to the Federation Nationale des Centres de Lutte Contre le Cancer (FNCLCC) classification [17]; clinical evolution; and treatment-related variables (type of surgery, chemotherapy and radiotherapy). Initial staging was based on chest and abdomen CT scans.

A multidisciplinary medical team validated all treatments according to local and national guidelines. Surgery was performed to achieve local control with tumor-free margins. Extent of tumor resection was evaluated from the surgeon’s notes, charts and pathological reports. Surgical resection was considered macroscopically complete (no visible tumor remaining) or incomplete (R2=gross tumor remaining post-operatively). Microscopically, tumor margins were defined as involved (R1=residual disease) or tumor-free (R0). Postoperative radiotherapy involved irradiation of all dissected tissues with a large field. Radiation therapy was administered at a dose of 50–66 Gy, from 1.8 to 2 Gy. Chemotherapy involved 6 cycles of doxorubicin, 60 mg/m^2^, delivered every 21 days. Ifosfamide, 2500 mg/m^2^, was given at days 1–3 for patients with performance status 0–1. Patients received doxorubicin and/or ifosfamide or another regimen, depending on their performans status and past medical history.

All patients were followed until death or the last known visit. Patients who underwent surgery were seen 1 month after hospital discharge. Every 3 months thereafter, physical and radiological examinations were performed. Recurrence was defined as tumor growth occurring at the excision site at least 3 months after the initial surgery (local recurrence) and/or new distant lesions (metastatic recurrence). Follow-up data included time to recurrence and type of recurrence (local and/or metastatic). Patients with advanced disease underwent physical examination before each chemotherapy cycle and CT scan every 3 cycles. Time to treatment failure was defined as time between diagnosis and recurrence.

### Statistical analysis

Descriptive variables (clinical, demographic, and biological) are represented with median (range) for continuous data and categorical variables with frequency (percentages) with 95% confidence intervals (95% CIs). Chemotherapy regimen (single versus double agent) was used to stratify time to treatment failure and overall survival analyses. Survival curves were plotted by the Kaplan-Meier method. We could not perform multivariate analysis because of the small sample size. Thus, we performed univariate regression analysis by the Cox proportional hazards model to explore the effect of explanatory variables such as anatomic location, age, tumor size, histological grade, gender and chemotherapy regimen on time to treatment failure and overall survival, estimating hazard ratios (HRs) and 95% CIs.

All statistical tests were two-sided with p < 0.05 considered statistically significant. Statistical analysis involved use of SPSS v15.1 (SPSS Inc., Cary, NC, USA) and EpiInfo v3.4.2 (Centers for Disease Control and Prevention, Atlanta, GA, USA).

## Results

**Figure 1:**
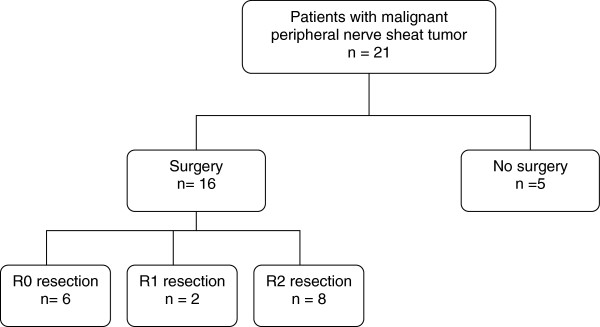
Flow-chart for surgery.

We included data for 21 patients (9 females; median age 31 years [range 18–79 years]). Patient characteristics are in Table 1. Primary tumors were located on extremities (n=8), abdomen or pelvis (n=6), trunk (n=4), and head or neck (n=3). The median tumor diameter was 13 cm [3–28 cm]. Pain, growing mass or neurological disorders was found in 20, 19 and 10 patients, respectively. FNCLCC histological grading of tumors was grade 1 (n=1, 4.8%), 2 (n=8, 38.1%) and 3 (n=12, 57.1%). At the time of initial diagnosis, 16 patients presented localized disease and were candidates for curative resection. However, only 8 underwent complete macroscopic resection. For these patients, tumor margins were classified as R1 (n=2) and R0 (n=6). The flow for surgery is presented in Figure 1. Three patients underwent amputation to achieve tumor-free margins.

**Table 1:** Patient and tumor characteristics for 21 patients with neurofibromatosis 1 and a diagnosis of malignant peripheral nerve-sheath tumors between 1993 and 2003

**Age, years**	
Median (range)	31 (18–79)
Tumor size, cm	
Median (range)	13 (3–28)
Gender, n (%)	
Male	12 (57)
Female	9 (43)
Metastases at diagnosis, n (%)	
Absent	18 (86)
Present	3 (14)
Type of surgery, n (%)	
R0, complete resection	1 (5)
R1, incomplete resection (microscopically)	3 (14)
R2, incomplete resection (macroscopically)	7 (33)
No surgery	10 (48)
Histological grade (FNCLCC), n (%)	
1	1 (5)
2	8 (38)
3	12 (57)
Pathological staging, n (%)	
T1	3 (14)
T2	18 (86)
Tumor site, n (%)	
Head and neck	3 (14)
Trunk	4 (19)
Extremity	8 (38)
Abdomen or pelvis	6 (29)
Chemotherapy, n (%)	
Single agent	6 (29)
Combination	15 (71)
Type of chemotherapy, n (%)	
Doxorubicin	5 (24)
Ifosfamide	1 (5)
Ifosfamide + anthracycline	11 (52)
Ifosfamide + anthracycline + platinum salt	4 (19)

Abbreviations: *FNCLCC* Federation Nationale des Centres de Lutte Contre le Cancer; *T* tumor.

**Figure 2:**
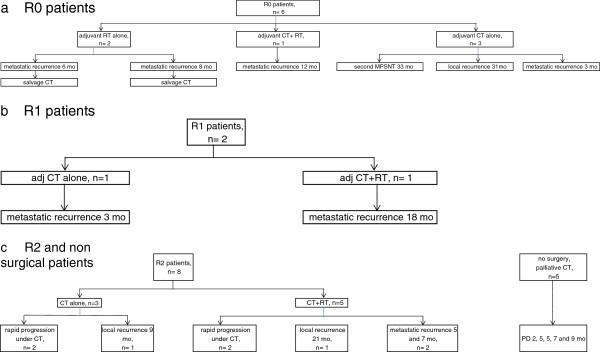
**Outcome of patients after initial treatment: a) R0 patients, b) R1 patients, c) R2 and non-surgical patients.** R2=macroscopic tumor remaining post-operatively, R1=microscopic involved margins (residual disease), R0= microscopic tumor-free margins.

For patients with R0 margins after resection, adjuvant treatment included chemotherapy (n = 3), radiotherapy (n = 2), or both (n = 1). Four of the 6 patients with R0 margins experienced metastatic recurrence at 3, 6, 8 and 12 months, respectively. Among the 4 patients who had received chemotherapy, 2 showed metastatic recurrence at 3 and 12 months, 1 patient showed local recurrence at 31 months (in a patient who had not received radiotherapy), and 1 showed contralateral MPNST at 33 months, followed by a third MPNST during follow-up (Figure 2a). This latter patient had undergone amputation for a 26-cm grade 3 tumor. The 2 other patients who underwent amputation died of metastatic disease.

The 2 patients with R1 margins after surgery received chemotherapy (n=2), with radiotherapy (n=1), but both experienced metastatic progression, at 3 and 18 months, respectively (Figure 2b).

Macroscopically incomplete resection (R2) was due to an internal location with large tumor size. The 8 patients with R2 status after surgery received post-operative palliative chemotherapy. Five also received radiotherapy that delivered a total dose of 50 Gy (n=4) or 30 Gy (n=1) before (n=3) or after the end (n=2) of the first-line chemotherapy. Four of the 8 patients showed rapid disease progression with chemotherapy.

Five patients did not undergo surgery and received only palliative chemotherapy. They showed progressive disease after 2, 5, 5, 7 and 9 months, respectively (Figure 2c).

All 21 patients showed treatment failure, with median time to treatment failure 7.8 months (range 2.1–128 months). At the time of the last follow-up (December 2012), 19 patients were dead, all due to cancer; 2 were still alive at 138 and 167 months, respectively, of follow-up. The first patient experienced 2 other MPNSTs and the second local recurrence, which was treated with surgery and radiotherapy. Both had localized grade 3 tumors with R0 resection (1 had an amputation) followed by chemotherapy early after surgery (6 and 32 days, respectively, as compared to a median of 101 days for the series). The chemotherapy regimen was ifosfamide and doxorubicin and was not associated with radiotherapy.

**Figure 3:**
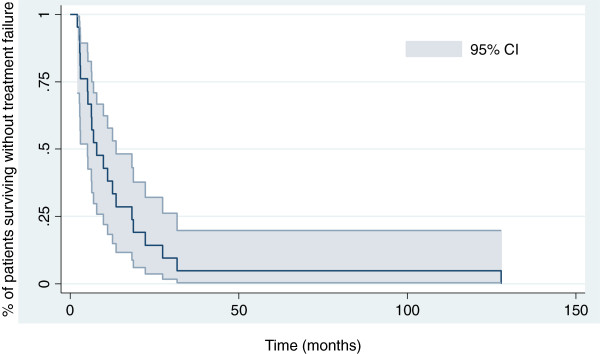
Kaplan-Meier curve for disease-free survival for 21 patients with malignant peripheral nerve-sheath tumors.

**Figure 4:**
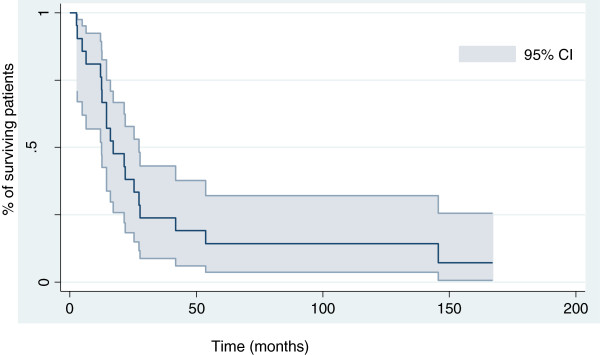
Kaplan-Meier curve for overall survival for 21 patients with malignant peripheral nerve-sheath tumors.

Survival at 12, 18 and 24 months was 81% (95% CI 76.4–86.6%), 47.6% (43–52.2%) and 38% (33.4–42.6%), respectively. The median overall survival for the entire cohort was 17 months (range 2.5–167 months) (Figures 3 and 4). The median time to survival for patients with peripheral MPNSTs was 21.4 months (range 14–137 months) and for patients with axial MPNSTs, 12.6 months (range 2.5–167 months, p=0.4). Univariate analysis revealed no association of variables examined and time to treatment failure or overall survival. Increased tumor size at diagnosis was associated with a short time to treatment failure (HR=2.7, 95% CI 0.9–7.8, p=0.07) and overall survival (HR 3.1, 1.1–8.9). As compared with locally advanced or metastatic disease, R0 and R1 status was associated with reduced risk of death [HR 0.24, 0.05–1.17; p=0.08]. Because of the small number of patients, we could not identify factors associated with the 2 amputation failures.

## Discussion

In our experience managing NF1 with MPNSTs by chemotherapy, overall survival was poor, with a median time of 17 months and 5-year survival of 14%.

Although a difference in survival between sporadic and NF1-related MPNSTs is still a matter of debate, several studies showed that the prognosis of patients with NF1 is poor [7,10,12,14,18]. This poor outcome can be explained by several points. For most of our patients, MPNSTs had a deep location, with large dimensions and high histological grade (poorly or undifferentiated tumors in 12 patients), as previously shown [11,12]. We reveal a low rate of complete surgery according to the localization and size of lesions at diagnosis: only 6 of the 21 patients had R0 resection, and local control rate was low. Indeed, most of the MPNSTs were internal (n=13) as previously shown [15]. In contrast, for patients with peripheral MPNSTs, survival was better, although not significantly, than with axial MPNSTs (median overall survival of 21.4 and 12.6 months, respectively; p=0.4). This finding can be explained by internal MPNSTs (in the abdomen, pelvis, chest, etc.) usually remaining asymptomatic until they reach a large size, whereas 5-year survival is better with MPNSTs < 5 cm [8,12]. Enlarged mass, neurological deficit and pain are clinical factors associated with malignant transformation [9]. These factors are more likely to be noted in peripheral than internal lesions.

Our series emphasizes the important role of surgery in the management of MPNST. Indeed, the 2 surviving patients underwent surgery with R0 margins, requiring amputation in 1. We were not able to identify any prognostic factor associated with survival in these patients, but we noted that both had received doxorubicin and ifosfamide early after surgery.

Regarding amputation, our data are consistent with literature: for the 3 patients who underwent amputation, only one survived. Non-conservative surgery is associated with better local control but not with better survival in these patients, as previously reported [13]. We lack a reliable prognostic factor of success for these non-conservative surgical strategies. Further studies should be performed to identify prognostic factors, and to evaluate the role of neo-adjuvant treatments. A study of neo-adjuvant isolated limb perfusion with tumor necrosis factor showed partial response in 3/4 patients with MPNST [19].

All our patients receiving chemotherapy experienced treatment failure. The place of chemotherapy in the management of NF1 with MPNSTs is still controversial [20]. In the adjuvant setting, chemotherapy is considered optional but is largely used (up to 40% of NF1 patients [18]), although doxorubin regimens have failed to show a benefit for local recurrence, distant recurrence, overall recurrence, and overall survival [21]. Adjunct therapy with ifosfamide might improve prognosis but with more toxicity.

Metastatic MPNSTs have poor prognosis, and all our patients receiving chemotherapy without surgery for advanced or metastatic diseases experienced disease progression. Chemotherapy is considered palliative in metastatic diseases [20]. Indeed, partial response rates are about 25% to 30% [8,22].

In our retrospective experience, alternative strategies, including targeted therapy, were considered. Significant advances in the pathophysiologic features of NF1 have led to considering this new therapeutic approach. MPNSTs present complex chromosomic alterations and additional genetic mutations (p53 and cyclin-dependent kinase inhibitor 2A) that are involved in malignant transformation [23]. Loss of *Nf1* gene expression induces lack of neurofibromin synthesis, a GTPase activating molecule that normally inactivates Ras and inhibits cell proliferation [24]. Aberrant activation of the Ras pathway in NF1 leads to cell proliferation. Furthermore, several signaling pathways involved in angiogenesis (cyclooxygenase 2, vascular endothelial growth factor A), cellular regulation (MKI67 etc.), epidermal growth factor (EGF receptor, pAKT, etc.) and Sonic hedgehog–Gli pathways are modified in plexiform neurofibromas associated with transformation [25].

Targeted therapies have had interesting results with NF1 tumors. Mammalian target of rapamycin inhibitors are considered a potential therapeutic approach [26-28]. More recently, preclinical studies have provided a rationale for testing mitogen-activated protein/endothelial regulated kinase inhibitors in NF1 clinical trials [29].

## Conclusions

MPNSTS are currently treated as other soft-tissue sarcomas, because they are too rare to perform trials with a sufficient number of patients. Overall survival with MPNSTS is poor, and the usual chemotherapy used for soft-tissue sarcomas does not improve the outcome. Recent advances in the molecular biology of MPNSTS may provide new targeted therapies.

## Abbreviations

CI: Confidence interval; FNCLCC: Federation Nationale des Centres de Lutte Contre le Cancer; HR: Hazard ratio; MPNSTs: Malignant peripheral nerve-sheath tumors; NF1: Neurofibromatosis 1.

## Competing interests

The author’s declare no competing interests.

No financial support was provided for this work from pharmaceutical industry or other institutions.

## Authors’ contributions

OZ made substantial contributions to the analysis and interpretation of data and was involved in drafting the manuscript. EF made substantial contributions to the acquisition, analysis and interpretation of data and was involved in drafting the manuscript. LZ made substantial contributions to the analysis and interpretation of data and was involved in revising the manuscript critically for important intellectual content. ES performed the statistical analysis, made substantial contributions to the analysis and interpretation of data and was involved in revising the manuscript critically for important intellectual content. NO performed histopathological analysis of the tumor specimens and was involved in revising the manuscript critically for important intellectual content. EB made substantial contributions to the acquisition of data and was involved in revising the manuscript critically for important intellectual content. PW made substantial contributions to the analysis and interpretation of data and was involved in revising the manuscript critically for important intellectual content. LVA made substantial contributions to the interpretation of data and was involved in drafting the manuscript. All authors have read and approved the final version to be published.
